# Permeability of Epithelial/Endothelial Barriers in Transwells and Microfluidic Bilayer Devices

**DOI:** 10.3390/mi10080533

**Published:** 2019-08-13

**Authors:** Timothy S. Frost, Linan Jiang, Ronald M. Lynch, Yitshak Zohar

**Affiliations:** 1 Department of Biomedical Engineering, University of Arizona, Tucson, AZ 85721, USA; 2 Department of Aerospace and Mechanical Engineering, University of Arizona, Tucson, AZ 85721, USA; 3 Department of Physiology, University of Arizona, Tucson, AZ 85721, USA

**Keywords:** barrier permeability, epithelial–endothelial interface, paracellular/transcellular transport

## Abstract

Lung-on-a-chip (LoC) models hold the potential to rapidly change the landscape for pulmonary drug screening and therapy, giving patients more advanced and less invasive treatment options. Understanding the drug absorption in these microphysiological systems, modeling the lung-blood barrier is essential for increasing the role of the organ-on-a-chip technology in drug development. In this work, epithelial/endothelial barrier tissue interfaces were established in microfluidic bilayer devices and transwells, with porous membranes, for permeability characterization. The effect of shear stress on the molecular transport was assessed using known paracellular and transcellular biomarkers. The permeability of porous membranes without cells, in both models, is inversely proportional to the molecular size due to its diffusivity. Paracellular transport, between epithelial/endothelial cell junctions, of large molecules such as transferrin, as well as transcellular transport, through cell lacking required active transporters, of molecules such as dextrans, is negligible. When subjected to shear stress, paracellular transport of intermediate-size molecules such as dextran was enhanced in microfluidic devices when compared to transwells. Similarly, shear stress enhances paracellular transport of small molecules such as Lucifer yellow, but its effect on transcellular transport is not clear. The results highlight the important role that LoC can play in drug absorption studies to accelerate pulmonary drug development.

## 1. Introduction

Drug discovery is becoming slower and more expensive over time, a trend referred to as Eroom’s law (Moore’s law spelled backward), despite major progress in technologies such as high-throughput screening and computational drug design [1]. Following this trend, the number of new drugs produced per billion US $ has halved every decade since 1950 [1,2]. The increasing difficulty and cost of developing a new compound can be traced back to current methods of new compound screening. In vitro cell cultures and animal models are the major means used to select promising drugs for clinical trials. Experience has shown that these models are poor predictors for compound success in human clinical trials. In vitro cell models in particular have had a limited impact, since they are overly simple failing to recreate the complex tissue microenvironment in vivo, especially the interactions between multiple cell types [3,4]. Animal models, while presenting in vivo tissue-level complexity, fail to provide the proper tissue microenvironment because animals do not possess the same anatomy or physiology as humans; consequently, these models have less than 8% successful translation to therapies in some cancer trials [5,6]. These serious shortcomings underline the critical need to develop new in vitro biomimetic systems that better represent the in vivo human physiological conditions in effort of hastening medical innovation [7].

Many pharmaceutical companies are turning to microfluidic devices as a means to streamline the pre-clinical phases of new compound screening and development [8,9]. Among this new generation of microfluidic devices are microphysiological systems (MPSs), often called organs-on-chips, which have begun to add additional insights as well as increased physiological accuracy to the screening of new compounds [10]. MPSs are in vitro models that capture important aspects of in vivo organ function through the use of specialized culture microenvironments [11]. These microfluidic systems, incorporating small plastic devices, are designed to model certain human organs to trigger more accurate cellular responses [12,13]. Indeed, MPS technology has begun to accelerate medical research across several fields. MPSs are used for modeling of diseases, such as cancer, which is emerging as a prominent application driving development of complex systems with higher-order tissue functions [11]. In drug discovery, these models are utilized to perform high-throughput assays to assess drug viability, optimizing clinical trials, and potentially reducing R&D costs to develop new compounds [14].

MPSs can be used to evaluate the entire life cycle of a compound inside a human body by culturing different human cell types in different chambers on the chip [15,16], and are often used to provide a unique window into the behavior of a tissue that cannot be observed in detail in vivo [17,18,19]. MPSs are particularly useful in exploring the dynamic permeability across barrier-tissue interfaces such as blood–brain, vascular-endothelial, and lung-blood barrier. Tissue barrier permeability is essential in the selective transport across the interface, is maintained through tight cell–cell junctions, and is controlled by growth factors, cytokines, and other stress related molecules [20]. Understanding the drug life cycle in the body begins with the absorption of a drug into the bloodstream. The intricate process of cellular transport is highly tailored to each organ and is dependent on the cell types being used as well as the expression of any proteins involved in the transport of a drug into the blood stream. Pulmonary drug delivery has recently become an attractive route for medical therapy as it is both minimally invasive and able to quickly interact with a large blood volume [21]. In order to study pulmonary absorption, static transwell inserts have been used as a standard assay for many years [22]. However, these static conditions lack the fluid-flow induced shear stress to mimic blood or air flow in human body. Media flow is an essential characteristic of lung-on-a-chip (LoC) devices allowing the introduction of mechanical forces in an effort to provide a more physiologically relevant model system [23]. Transport of bio-species ranging from small molecules such as water and ions, to large proteins, and even whole cells is significantly more complex in systems with media flow. On top of diffusion, convection is added as another mass transport mechanism. Furthermore, the shear stress exerted on the cellular layer may modify the tissue barrier properties through alteration of inter-cellular junctions and cellular interactions with the extracellular matrix [24,25,26]. With the growing interest in microfluidic pulmonary models to study drug absorption, a thorough evaluation of the lung–blood barrier is needed. Recently, some permeability measurements were reported in a small airway-on-a-chip model [23], and vascular-endothelial barrier permeability was characterized using a biomimetic microfluidic blood vessel model [27]. To date, little has been done to analyze the transport of larger molecules within an organ-on-a-chip device. In this work we utilize microfluidic membrane bilayer devices, typically used for LoC applications, to form epithelial/endothelial cell barriers. The permeability of various molecules, via different transport pathways, is characterized with and without epithelial/endothelial monolayers or bilayers, and the results are compared with corresponding data collected in traditional transwell inserts. In the current work, both epithelial and endothelial cell layers are submerged in liquid media although, for human lung models, an air-liquid interface is an important feature affecting molecular diffusion in the epithelial side of the interface and subsequently the permeability.

## 2. Methods

Two experimental setups have been utilized in this work: (i) commercially available transwell inserts under static condition to eliminate the effect of shear stress, and (ii) in-house fabricated microfluidic devices under fluid flow accounting for flow-induced shear stress. Epithelial and endothelial cell layers were cultured in the transwells inserts and microfluidic devices to characterize the permeability of various bio-species based on their concentration measurements. The experimental error analysis associated with these measurements has been reported elsewhere [28].

### 2.1. Transwell Insert Models

Either epithelial or endothelial cells were cultured in the top compartments (often called apical or luminal) of commercially-available 12-well transwells (Corning, Corning, NY, USA) on polyester membranes with 0.4 µm diameter pores and 0.5% porosity. Once the cultured cell monolayers had reached confluency, media in the top compartments were replaced with 500 µL solutions of cell media mixed with various fluorescently-labeled molecules at pre-determined initial concentrations. After filling the bottom compartments (often called basolateral or abluminal) with 1500 µL fresh cell media, the transwells were placed in an incubator for two hours. Then, liquid samples of 100 µL were removed from both compartments, 5 from the top and 15 from the bottom, and the corresponding fluorescent intensities of all samples were measured using a BioTek Synergy 2 Plate Reader (BioTek, Winooski, VT, USA). Molecular concentrations in the collected samples were then estimated based on the fluorescent intensity measurements using pre-established calibration curves. The average value among the samples collected from each compartment was taken as the molecular concentration. The experiments were repeated without cell monolayers in the transwells. Both the initial concentration and incubation time were determined to keep the concentration measurements within the linear range of the calibration curves to avoid signal saturation.

### 2.2. Microfluidic Devices

**Figure 1:**
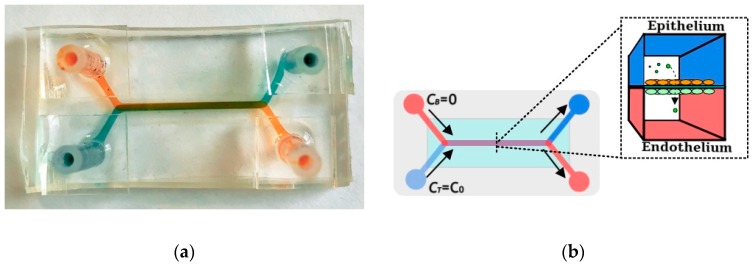
A microfluidic bilayer device: (**a**) A photograph of a fabricated and packaged microfluidic bilayer device with blue dye in the top and red dye in the bottom microchannel, and (**b**) a schematic of a microfluidic bilayer device in operation; the top epithelial microchannel is separated from the bottom endothelial microchannel channel by a porous membrane. A syringe pump is used to drive cell medium mixed with fluorescent molecular markers through the top channel and only cell medium through the bottom channel at equal rates; the fluorescent markers are transported across the membrane, from the top to the bottom microchannel and downstream along both microchannels.

Microfluidic devices were fabricated using soft-lithography techniques. Polydimethylsiloxane (PDMS, Sylgard 184 Silicone Elastomer, Dow Corning, Midland, MI, USA) resin was poured over an aluminum mold and allowed to cure for 24 h at 55 °C. The cured PDMS substrate with grooves, each about 35 mm long and cross-section area of 500 µm × 1000 µm, was peeled off the aluminum mold. A polyester track etched membrane, about 20 µm thick with 1% porosity and 0.8 µm diameter pores, was plasma treated and soaked in a solution of 5% (v/v) (3-Aminopropyl)triethoxysilane (APTES, MP Biomedicals, Solon, OH, USA) in water at 55 °C for 1 hour for leakage-free sealing [29]. While the membrane was drying, pairs of microchannel grooves were oxygen-plasma treated for 60 s at 1000 mTorr (Harrick Plasma, Ithaca, NY, USA). Each pair of PDMS microchannels, coated to prevent small molecule absorption [30], was bonded together with a membrane sandwiched between the overlapping channel segments about 20 mm in length. Once firm bonding had been achieved, tubing adapters were placed over the punched holes to serve as inlet/outlet connectors to the external fluid handling system. An image of a fabricated device along with a schematic of a device operation is shown in Figure 1.

### 2.3. Cell Cultures

Epithelial and endothelial cell lines were chosen to represent human lung–blood barrier interface. The epithelium was composed of carcinoma alveolar epithelial cells (A549; ATCC Manassas, VA, USA), cultured in RPMI media supplemented with 10% fetal bovine serum (FBS, Gibco, Waltham, MA, USA) (v/v) and 1% penicillin streptomycin (Sigma Aldrich, St. Louis, MO, USA). The A549 cell line has characteristic features of pulmonary epithelium, which has a role in the oxidative metabolism of drugs in the lung. This cell line has indeed been extensively utilized in toxicology studies, including its properties on permeable supports, because of its potential target for drug delivery of macro-molecules [31].The endothelium was composed of human umbilical vein cells (HUVEC; ATCC Manassas, VA, USA), cultured in F-12K media (Kaighn’s modification, Gibco, Waltham, MA, USA) with 10% FBS, 1% penicillin streptomycin, 50 µg/mL endothelial cell growth supplement (ECGS, Sigma Aldrich, St. Louis, MO, USA) from bovine neural tissue, and 100 µg/mL heparin (Sigma Aldrich, St. Louis, MO, USA). HUVEC cells were selected for this characterization phase of LoC models, since they are most commonly utilized to represent an endothelium based on human derived primary endothelial cells [32]. The cell passage number of the A549 and HUVEC culture was 40–60 and 20–30, respectively. Cell media inside the transwells and microfluidic devices was replenished every 48 h. All cell cultures were maintained at 37 °C, 5% CO_2_, 95% air, and 100% relative humidity.

Epithelial and endothelial cells were seeded in either transwells or microfluidic devices at a density of 2 × 10^6^ cells/mL. HUVEC cell attachment and proliferation required membrane surface coating with collagen. A 200 µg/mL type 1 collagen solution (Gibco, Waltham, MA, USA) was prepared in 0.01 M acetic acid and perfused across the membrane surface. After one hour of curing at an ambient temperature, the collagen solution was replaced with 1x HBSS followed by 30 min incubation time in the cell culture environment to adjust the system pH to a biocompatible level. The HUVEC media for cells cultured in transwell or microfluidic devices had an elevated ECGS concentration (150 µg/mL) to promote a fully confluent monolayer [33].

### 2.4. Tracer Molecules

**Figure 2:**
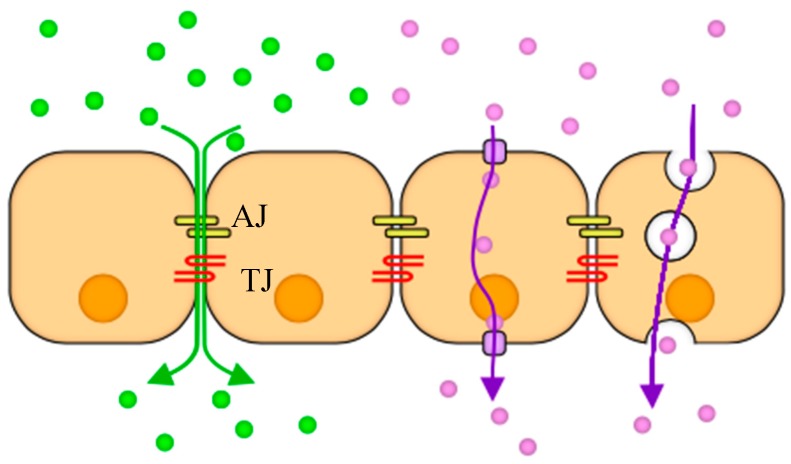
A schematic illustrating two cellular transport mechanisms: Paracellular (green) occurs as molecules diffuse through adherens (AJ) and tight junctions (TJ) between cells because of concentration gradient, and transcellular (purple) takes place when cells uptake and release molecules through their bodies via transport proteins or as vesicles.

Molecules can be transported through confluent cell monolayers via two major routes paracellular and transcellular as illustrated in Figure 2. A confluent monolayer refers to cells in culture, which are in contact forming a cohesive sheet of adhering neighboring cells. It is thought that contact inhibition of proliferation is a characteristic of a confluent monolayer, where cells stop proliferating upon contact formation [34]. Adherens junctions (AJs) and tight junctions (TJs) comprise two modes of cell–cell adhesion that provide different functions. While AJs initiate cell–cell contacts and mediate the maintenance of the contacts, TJs regulate the paracellular pathway for the movement of ions and solutes in-between cells [35]. In contrast, transcellular transport involves the passage of solutes through the cells crossing both the apical and basolateral membranes. The transcellular transport includes passive diffusion through ion channels, active carrier mediated transportation such as organic anion/cation transporters, and transcytosis of macromolecules such as transferrin and insulin [36]. Various fluorescently labeled molecules were used to study both molecular pathways in the transwells and in the microfluidic devices.

Fluorescently labeled tracer molecules commonly used in permeability studies were chosen as biomarkers for paracellular and transcellular transport. Lucifer yellow (0.44 kDa; Invitrogen, Carlsbad, CA, USA) has previously been used as a small molecule paracellular transport marker [31,37]. However, it should also be noted that a growing body of literature suggests that transcellular passage of Lucifer yellow may also occur via organic anion transporters [38]. Dextrans, non-digestible sugars, were selected to represent typical agents that are transported via the paracellular route through cell tight junctions [27,39]. Expression by epithelial or endothelial cells of membrane receptors mediating transcytosis of dextran has not been reported thus far; however, pinocytosis of dextran was observed [40], in which molecules are captured in vesicles on one side of the cell, drawn across the cell, and ejected on the other side. Since pinocytosis is a very slow process, transcellular transport of dextran is negligible compared to its paracellular transport [41]. To explore potential molecular-size effect, 4-dextran (4 kDa) and 70-dextran (70 kDa) (Invitrogen, Carlsbad, CA, USA) were chosen to represent the intermediate and large-size agents, respectively. FITC-transferrin (78 kDa; Rockland Antibodies, Limerick, PA, USA) was used to represent a typical protein that is transported via the transcellular route, transcytosis, where epithelial A549 and endothelial HUVEC cells uptake the molecules by binding transferrin to its membrane receptor expressed by both cell lines [31,39,42,43]. FITC-transferrin and 70-dextran molecules are about the same in size and, therefore, may share similar paracellular transport level.

### 2.5. Concentration Measurements in Microfluidic Devices

Epithelial or endothelial cell monolayers were cultured with their specific medium, described in Section 2.3, on the separation membranes in the top channels. Co-cultures of cell bilayers were constructed by growing epithelial and endothelial cells on opposite surfaces of the separation membranes. HUVEC cell suspensions were first seeded with endothelial medium in the top channels on collagen-coated surface membranes. Once the HUVEC cell monolayers reached full confluency, typically within 1–2 days, the bottom channels were perfused with suspensions of A549 cells in the endothelial medium. The tubing adapters were temporarily plugged, and the microfluidic devices were flipped upside down. The inverted devices were suspended over a small water bath for 24 h allowing the establishment of epithelial and endothelial confluent monolayers. The devices were then flipped back to an upright position and the tubing adapters were un-plugged. Live cell staining was performed using CellTracker fluorescent probes (Invitrogen, Carlsbad, CA, USA), within the microfluidic devices, and cells were immunostained for known intercellular adhesion biomarkers to confirm confluency of the cell layers.

In all cases, with or without cells, the top and bottom channels were filled with cell media solution mixed with various tracer molecules at pre-determined initial concentrations and cell media with zero molecular concentration, respectively. Both microchannels were then connected to a PHD Ultra syringe pump (Harvard Apparatus, Holliston, MA, USA) to drive the media in each channel at the same constant flow rate to minimize trans-membrane pressure differences. A549 cell medium was flown through both channels only when epithelial monolayers were tested. In all other conditions, including cell bilayers, HUVEC cell medium was flown through both channels. The temporal fluorescent intensities because of decreasing concentrations at the top and increasing concentrations at the bottom microchannel outlets, with and without cells, were recorded at five minutes intervals. The channel outlets were exposed to a light source for a short time, less than 5 s, to limit photo bleaching, and calibration curves were used to relate the measured fluorescent intensities to molecular concentrations. For steady-state experiments, similar to the transient experiments, microfluidic devices with and without confluent cell monolayers or bilayers were connected to the syringe pump under several constant flow rates. Following the development of steady-state molecular concentration distributions, 500 µL effluent samples from both microchannels were collected and the corresponding fluorescent-light intensities were measured using the BioTek Synergy 2 well plate reader. The sample collection time was 25, 10, 5, and 2.5 h for 20, 50, 100, and 200 µL/h flow rate, respectively, with the microfluidic devices placed in while the syringe pump kept outside of an incubator. As in the transwell experiments, the same calibration curves were used to relate the light intensities to molecular concentrations. The initial inlet concentrations in the microfluidic temporal and steady experiments were the same as those applied in the transwell experiments. It was important to determine the time to reach steady state from the temporal experiments to ensure collection of effluent samples under steady-state conditions. The tested flow rate range was 20–200 µL/h with a corresponding wall shear stress range of 0.0012–0.012 dyne/cm^2^.

## 3. Results and Discussion

**Figure 3:**
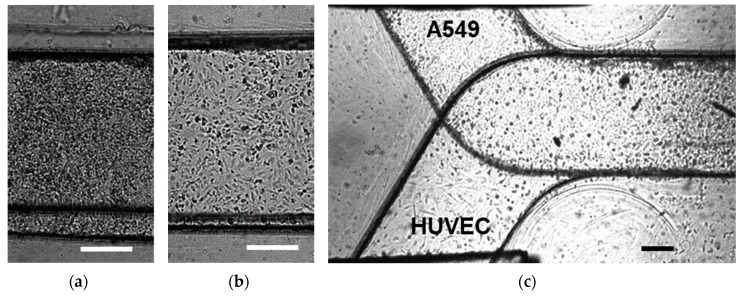
Microscopic bright-field images of: (**a**) confluent A549 epithelial cell monolayer, (**b**) confluent human umbilical vein cells (HUVEC) endothelial cell monolayer, and (**c**) confluent A549/HUVEC cell bilayer cultured in microfluidic devices; scale bars are 250 μm.

A549 and HUVEC cell monolayers as well as A549/HUVEC bilayers were successfully cultured in microfluidic devices as shown by the bright-field images in Figure 3a–c. Since it is difficult to distinguish between different cell types in bright-field images of bilayers cultured on opposite surfaces of a membrane, live cell staining was performed within a microfluidic device to confirm successful establishment of an A549/HUVEC cell co-culture.

**Figure 4:**
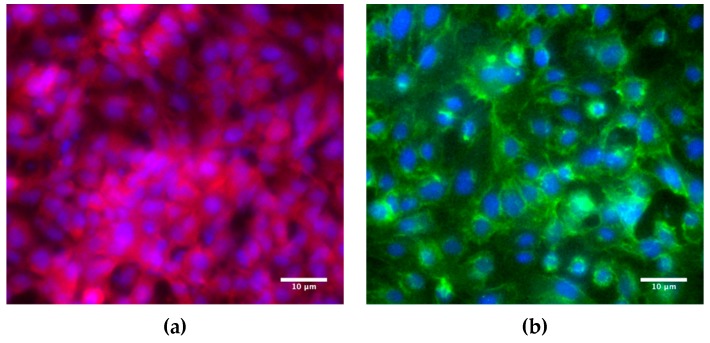
Fluorescence microscope images of: (**a**) A549 epithelial cells immunostained for ZO-1 cytoplasmic proteins (red) as a biomarker for tight junctions, and (**b**) HUVEC endothelial cells immunostained for VE-cadherin transmembrane proteins (green) as a biomarker for adherens junctions with standard Hoechst staining (blue) of both A549 and HUVEC cell nuclei. The images were taken from slide-mounted membranes, which were removed from the microfluidic devices following cell fixing and staining.

Cell layer confluency is typically verified via staining particular proteins involved in cell–cell adhesion. Previous work has suggested that A549 form tighter barriers with the presence of intercellular junction proteins [44,45]. Tight junctions comprise transmembrane proteins (occludin, claudins, JAM), cytoplasmic attachment proteins (ZO-1, ZO-2, ZO-3), and cytoskeleton protein F-actin [46]. The ZO-1 cytoplasmic protein was selected for staining as a biomarker for tight junctions in the A549 cells. The abundance of the red signal in Figure 4a, corresponding to the stained ZO-1 proteins (Proteintech, Rosemont, IL, USA), is evidence for the formation of tight junctions and confluency of the A549 cell monolayer. Adherens junctions in endothelial cells are mainly composed of cadherins and catenins [47]. The vascular endothelial cadherin (VE-cadherin, Cell Signaling Technology, Beverly, MA, USA), a calcium-dependent cell–cell adhesion transmembrane glycoprotein, was selected for staining as a biomarker for adherens junctions in the HUVEC cells. The green signal in Figure 4b, corresponding to the stained VE-cadherins, demonstrates the formation of Adherens junctions and confluency of the HUVEC cell monolayer.

### 3.1. Molecular Transport in Transwells

Diffusion in transwells is a process continuing until an equilibrium state is reached, at which the concentration at the bottom equals the concentration at the top compartment with zero gradient. The parameter used for assessing molecular transport is the relative temporal concentration at the bottom compartment, *C*(*t*), defined as:

(1) $$\frac{C\left(t\right)}{C_{0}}=\frac{C_{B}\left(t\right)}{C_{T}\left(t\right)+3\cdot C_{B}\left(t\right)}$$

where *C_B_*(*t*) and *C_T_*(*t*) are the average molecular concentrations measured at the bottom and top compartments, respectively, with initial conditions *C_B_*(*t* = 0) = 0 and *C_T_*(*t* = 0) = *C*_0_. Initial concentrations for Lucifer yellow, 4-dextran, 70-dextran, and transferrin molecules were *C*_0_ = 100, 60, 1, and 1 µM, respectively. Since the bottom compartment volume is three times larger than the top compartment volume, the equilibrium concentration is expected to be a quarter of the top initial concentration, i.e. *C_B_*(*t*→∞) = *C_T_*(*t*→∞) = *C*_0_/4, independent of molecule or cell type. Therefore, to elucidate the effect of different cell monolayers on the transport of various molecules, experiments were terminated after two hours to ensure that the concentration at the bottom compartment, while high enough to allow reliable measurements, is still smaller than the equilibrium level.

**Figure 5:**
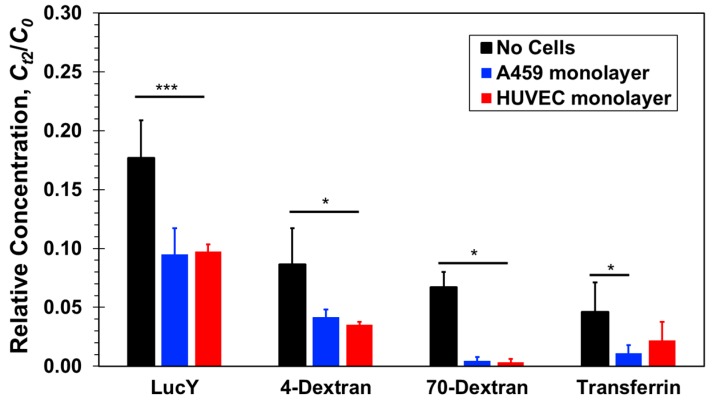
Relative concentrations of four molecular markers, Lucifer yellow, 4-dextran, 70-dextran, and transferrin, measured at the bottom compartments of transwells with and without confluent A549 epithelial or HUVEC endothelial cell monolayer after a 2-h incubation time. Significance determined by Student’s *t*-test; *P < 0.05; ***P < 0.001; *n* > 3.

The measured relative concentrations of three paracellular-transport markers, Lucifer yellow, 4-dextran, and 70-dextran, and one transcellular-transport marker, transferrin, are compared in Figure 5 with and without confluent cell monolayers. All relative concentrations measured at the bottom compartments are smaller than the equilibrium concentration, *C_t_*_2_ = *C*(*t* = 2 h) < 0.25*C*_0_, indicating that the diffusion process is still ongoing after the two-hours incubation time. Without cells, black columns, the relative concentration decreases with increasing molecular size. Based on the Stokes–Einstein equation, molecular diffusivity depends on its size as follows [48,49]:

(2) $$D=\frac{k_{B}\cdot T}{6\pi \mu r}\;\mathrm{with}\;r={\left(\frac{3M}{4\pi \rho N}\right)}^{1/3}$$

where *k_B_* is the Boltzmann constant, *T* is the temperature, *μ* and *ρ* are the fluid viscosity and density, respectively; *r* is a radius calculated based on the molecular weight, *M*, and the Avogadro number, *N*. Following the convection-diffusion equation:

(3) $$\frac{\partial c}{\partial t}+u\cdot \nabla c=D\nabla^{2}c$$

The spatiotemporal concentration is directly proportional to the molecular diffusion coefficient, *D*, where **u** is the velocity vector. Lucifer yellow is the smallest molecule in this study with the highest diffusion coefficient, 5.0 × 10^−10^m^2^/s, while the diffusion coefficients of the large 70-dextran and transferrin molecules are much smaller, 5.6 × 10^−11^m^2^/s and 5.85 × 10^−11^m^2^/s, respectively, and the diffusion coefficient of 4-dextran is intermediate about 1.35 × 10^−10^m^2^/s. Thus, in the absence of cells, the measured relative concentration decreases because of decreasing diffusivity with increasing molecular size.

In the presence of cell monolayers, either epithelial A549 or endothelial HUVEC, the measured relative concentrations, *C_t_*_2_/*C*_0_, are smaller than those measured in the absence of cells (Figure 5). Lucifer yellow and both dextran molecules have been widely considered as paracellular markers [27,31]. Hence, their mass transfer rate is generally not aided by active membrane transport proteins; it is rather a passive diffusion through the cellular junctions and membrane pores due to local concentration gradients. The total mass transferred from the top to the bottom compartment, and the resulting bottom concentration, is the integral over time of the product of molecular flux and flux area. Therefore, since the cell monolayers present additional resistance connected in series with the membrane resistance to molecular transport through the combined barrier, the molecular flux decreases in the presence of cells and with it the molecular concentration. Furthermore, in the case of the large 70-dextran, the relative concentration with cells is at the background level suggesting that the confluent monolayers essentially blocked molecular transport between the two compartments. For the smaller molecules, the concentrations measured in the presence of cells were found to be about half of the measured concentrations without cells but clearly above background levels. Since neither A549 nor HUVEC cells are known to uptake these molecules, they penetrated either one of the confluent monolayers via the paracellular route. The junctions of the confluent A549 and HUVEC cell monolayers appear to be tight enough to block the transfer of large proteins but leaky to small proteins amounting to a reduction of the separation-membrane porosity. HUVEC cell layers are known to be leaky with low paracellular resistance [50], and the leaky junctions are associated with cell proliferation or turnover (mitosis) and cell death (apoptosis) [18,51]. A549 cell layers have also been reported to be much leakier than other conventional epithelial cell lines [52]. Indeed, the leakiness of A549 cell monolayers limits their utility in examining the transport of low molecular weight drugs [31]. Therefore, it is not surprising that for these three paracellular markers, no clear difference has been observed between paracellular transport through a confluent endothelial HUVEC and an epithelial A549 monolayer.

The size and diffusivity of FITC-transferrin and large 70-dextran are about the same. Therefore, in the absence of an alternative transport mechanism, paracellular transport of both molecules through a confluent cell monolayer should be similar. Indeed, the transferrin concentration measurements with cells are smaller than the measured concentration without cells. However, while the concentration level of 70-dextran with cells is nearly zero, the measured concentrations of transferrin are statistically significant above background level. Assuming that a confluent monolayer of either A549 or HUVEC cells renders paracellular transport of transferrin negligible, similar to 70-dextran, the elevated transferrin concentration measurements can be attributed to the transcellular transport not available for dextran molecules. Hence, it seems that transferrin receptors expressed by both A549 and HUVEC cells actively facilitate transcellular transport of transferrin molecules not through the tight junctions of the confluent monolayers. The transferrin relative concentration with HUVEC cells is about double that with A549 cells, suggesting either higher transport rate or higher receptor density in HUVEC cells.

### 3.2. Molecular Convection–Diffusion in Microfluidic Devices

The convection–diffusion experiments in the microfluidic device start with initial conditions of zero molecular concentration at the bottom channel inlet and a uniform concentration at the top channel inlet, *C*_0_. Molecular transport from the top to the bottom channel starts after imposing the same constant flow rate through both channels. The time-dependent molecular concentrations are estimated from the average concentration measurements, *c*(*x*,*y*,*z*;*t*), which were recorded at the top and bottom channel outlets yielding:

(4) $$C_{T}\left(t\right)=\left[\overset{W/2}{\underset{-W/2}{\int }}\overset{H+h}{\underset{h}{\int }}c\left(x=L/2,y,z;t\right)dydz\right]/\left(W\cdot H\right)$$

and

(5) $$C_{B}\left(t\right)=\left[\overset{W/2}{\underset{-W/2}{\int }}\overset{-h}{\underset{-H-h}{\int }}c\left(x=L/2,y,z;t\right)dydz\right]/\left(W\cdot H\right)$$

where *H*, *W*, and *L* are the channel height, width, and overlapping length, respectively, and *h* is half the membrane thickness. The origin of the coordinate system is chosen to be at the center of the device such that the fluid domain is confined to the top: −*L*/2 ≤ *x* ≤ L/2, *h* ≤ *y* ≤ *H+h*, −*W*/2 ≤ *z* ≤ *W*/2, and bottom channel: −*L*/2 ≤ *x* ≤ L/2, −*H*−*h* ≤ *y* ≤ −*h*, −*W*/2 ≤ *z* ≤ *W*/2. Because of the convection effect, a steady-state concentration distribution is developed before complete mixing of the two fluid flows such that *C*_∞_ = *C_B_*(*t*→∞) < *C*_0_/2. Initial concentrations in microfluidic devices were identical to those in the transwell experiments, i.e., *C*_0_ = 100, 60, 1, and 1 µM for Lucifer yellow, 4-dextran, 70-dextran, and transferrin molecules, respectively.

#### 3.2.1. Transient Response Characterization

**Figure 6:**
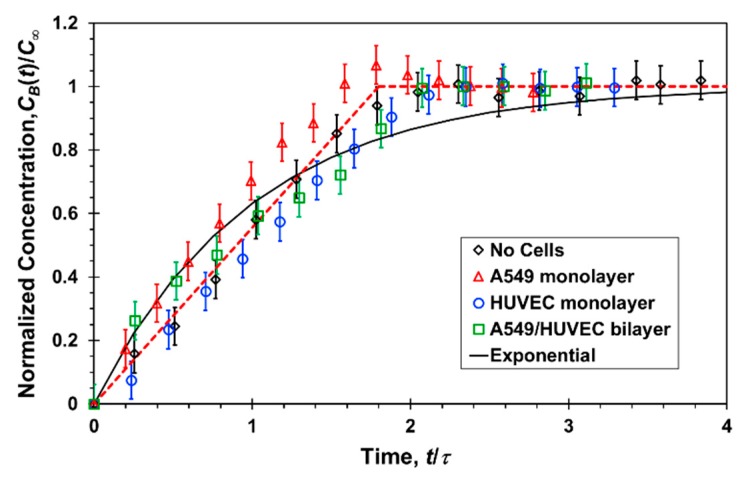
Normalized Lucifer yellow concentration measured at the bottom channel outlet of a microfluidic device as a function of time, under a 20 µL/h flow rate in each channel, with and without confluent cell mono/bilayers; the experimental data (symbols) are fitted with a linear (red dash line) and exponential function (black solid line).

The temporal measurements of Lucifer yellow concentrations at the bottom channel outlets of the microfluidic devices with and without cells, under a flow rate of 20 µL/h, are summarized in Figure 6. The measured concentrations are normalized by the steady-state concentration, *C*_∞_ = 0.2*C*_0_, and a characteristic time scale of *τ* = 25 min. In all cases, the system had reached a steady state within one hour. More importantly, the collapse of the data indicates that the bottom-channel concentration increase with time, *C_B_*(*t*), in the absence of cells is very much the same as in the presence of cells, either an A549 or HUVEC monolayer or an A549/HUVEC bilayer. Thus, within experimental error, each of the three cell-culture combinations has negligible effect on the transport rate of Lucifer yellow molecules from the top to the bottom channel. In contrast, in the transwell static experiments, the concentration at the bottom compartment with cells was found to be about half of that with no cells, and is discussed next.

#### 3.2.2. Steady-state Molecular Transport

**Figure 7:**
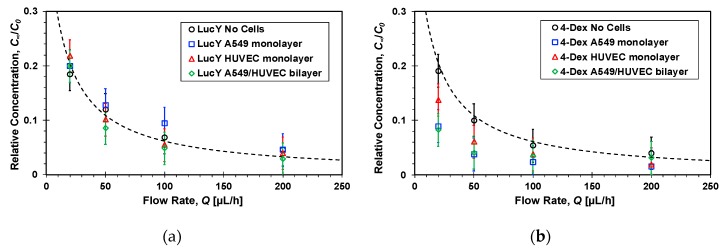

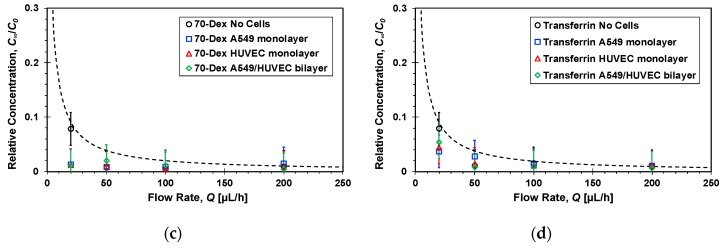
Flow rate effect on the steady-state relative concentrations of: (**a**) Lucifer yellow, (**b**) 4-dextran, (**c**) 70-dextran, and (**d**) transferrin markers measured at the bottom channel outlets of microfluidic devices, because of molecular transport from top to bottom channel, with and without confluent cell mono/bilayers; dash lines are functions inversely proportional to the flow rate fitted to the no cell experimental data with *R*^2^ = 0.9.

The flow rate, *Q*, is the most dominant parameter controlling the molecular transport rate between the two channels as it can vary from a very low rate resulting in complete mixing of the two streams, i.e., *C*_∞_→*C*_0_/2 as *Q*→0, to a flow rate high enough to render cross-membrane diffusion negligible such that *C*_∞_→0 as *Q*→∞. Therefore, the average molecular concentration at the bottom channel outlet was measured under different flow rates once steady state concentration distributions had been established within the microfluidic devices. The relative concentrations of the four molecules, *C*_∞_/*C*_0_, in the absence and presence of cell cultures are detailed in Figure 7 as a function of the flow rate. In general, in all cases with and without cells, the concentration is roughly inversely proportional to the flow rate decreasing with increasing flow rate. Under high flow rate, *Q* > 100 µL/h, the measured concentrations are so low such that it is very difficult to elucidate the effect of the cell cultures on the ensuing molecular transport because of the large experimental error. Phase contrast imaging revealed no changes in the confluence or morphology of monolayers cultured under the tested flow rate range, *Q* = 30–200 µL/h.

**Figure 8:**
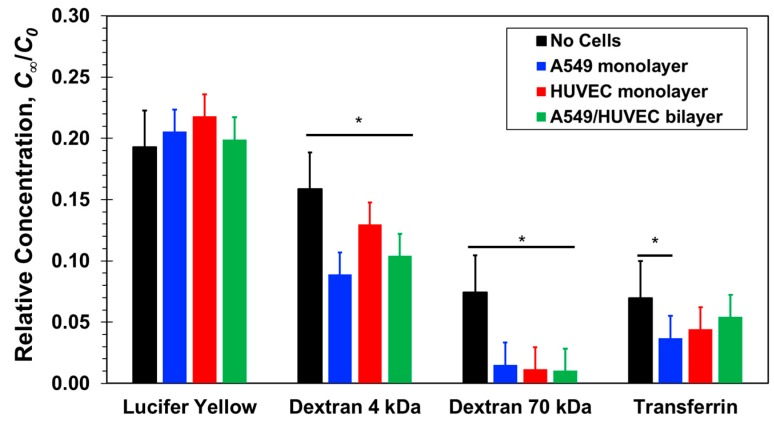
Steady-state relative concentrations of four molecular markers, Lucifer yellow, 4-dextran, 70-dextran, and transferrin, measured at the bottom channel outlets of microfluidic devices with and without confluent cell mono/bilayers under a 20 µL/h flow rate in each channel. Significance determined by Student’s *t*-test; *P < 0.05; *n* > 3.

The steady concentrations measured at the bottom channel outlets under a 20 µL/h flow rate, however, are high enough to facilitate a meaningful discussion and are depicted in Figure 8. In microfluidic devices, similar to the results in the transwells, the concentrations of the 4-dextran molecules in the presence of cells are smaller than the concentration without cells; namely, in both cases, leaky junctions of the confluent cell layers failed to block paracellular transport of intermediate-size molecules but rather presented an added resistance akin to decreasing membrane porosity. Also, the concentrations of the 70-dextran in the presence of cells are within the background noise indicating that the cellular junctions are tight enough to prevent paracellular transport of large molecules. On the other hand, in contrast with the transwells findings, the Lucifer yellow relative concentrations with and without cells are about the same within experimental error. Since Lucifer yellow has been utilized as a paracellular marker, its uptake by A549 and HUVEC cells is assumed to be negligible. Thus, if indeed its transcellular transport is insignificant, it seems that the shear stress enhances paracellular transport of Lucifer yellow to such a level practically diminishing the resistance of the cell layers to small-molecule transport.

The concentration of transferrin is significantly larger than that of 70-dextran. Both molecules are similar in size and, since applied shear stress had no effect on transport of large 70-dextran molecules through cellular junctions, paracellular transport of transferrin is also expected to be negligible. Therefore, the measured transferrin concentration can be attributed to molecular transport via the transcellular route, i.e., transcytosis, as was observed in transwells. It is difficult to discern the contributions of the different cell layers to the transferrin transport because of the relatively large experimental error. However, in comparison with the transwells results, the small difference between transferrin concentrations with and without cells suggest that the shear stress in microfluidic devices can perhaps enhance transferrin transcellular transport by the active protein transporters.

### 3.3. Barrier Permeability Characterization

Barrier-tissue interfaces are semi-permeable allowing selective transportation of molecules such as water, ions, and nutrients across the interface. Such a permeability barrier is maintained through cell–cell junctions and is controlled by growth factors, cytokines, and other stress-related molecules. Barrier permeability is a dynamic process influenced by many factors including exposure to inflammatory agents and gene products. Permeability is a measure of the average molecular flux across a barrier interface area. In microfluidic devices, under steady-state conditions, the apparent permeability is given by:

(6) $$P_{am}=\left(C_{\infty }/C_{0}\right)\left(Q/A\right)$$

where *A* is the barrier interface area. However, in transwells, experiments are terminated prior to reaching an equilibrium of uniform concentration in top and bottom compartment; therefore, the permeability is calculated as follows:

(7) $$P_{at}=\left(C_{t2}/C_{0}\right)\left(V/A\right)\left(1/t\right)$$

**Figure 9:**
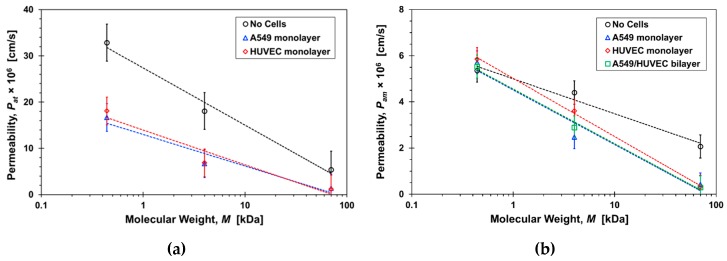
Molecular size effect on the permeability of paracellular markers, Lucifer yellow, 4-dextran, and 70-dextran, with and without cells in: (**a**) transwells after 2-hrs incubation time, *P_at_*, and (**b**) microfluidic devices under a 20µL/h flow rate, *P_am_*. Dash lines are logarithmic functions fitted to the corresponding data sets with *R*^2^ > 0.9.

*C_t_*_2_ is the measured concentration at the bottom compartment with a volume of *V* = 1500 µL following diffusion time of *t* = 2 h. The permeability values estimated based on Equations (6) and (7) in the range of 10^−6^–10^−5^ cm/s are in close agreement with previously published data [12,23,27,31]. The molecular size effect on the permeability, mainly because of paracellular transport, is demonstrated in Figure 9. The estimated permeability in transwells after two hours of diffusion (Figure 9a) and in microfluidic devices at steady-state under a 20 µL/h flow rate (Figure 9b) are plotted as a function of the molecular weight. The separation membrane permeability—without cells—is inversely proportional to the molecular size because of decreasing diffusivity with increasing size. The barrier permeability—with cell monolayers cultured on the separation membrane—decreases roughly logarithmically with increasing molecular size within experimental error. In static transwells, with no shear stress, the permeabilities of all three paracellular markers with a confluent A549 or HUVEC cell monolayer are markedly smaller than without cells; no clear difference between the epithelial- and the endothelial-monolayer permeability can be discerned. The trends in microfluidic devices with shear stress are similar to those in transwells, except for the Lucifer yellow permeability in microfluidic devices which is about the same with and without the cell layers. The permeability of the 70-dextran is close to zero in both systems. Since its transcellular transport is negligible, its paracellular transport through the cell TJs is also negligible because of its large size. Transcellular transport of 4-dextran is assumed to be negligible, similar to 70-dextran, independent of dextran molecular size. Therefore, because of its smaller size, the 4-dextran permeability in both transwells and microfluidic devices is likely due to paracellular transport through leaky TJs.

**Figure 10:**
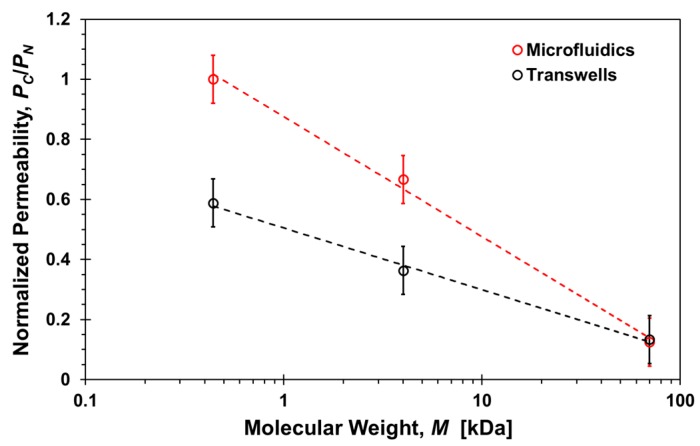
Molecular size effect on the ratio between the permeability with and without cells, *P_C_*/*P_N_*, of paracellular markers, Lucifer yellow, 4-dextran, and 70-dextran, in transwells after 2-h incubation time and microfluidic devices under a 20 µL/h flow rate. Dash lines are logarithmic functions fitted to the corresponding data sets with *R*^2^ > 0.9.

The effect of shear stress on permeability can be delineated from comparing the transwells with the microfluidic results. However, the microfluidic permeability was estimated under steady molecular flux independent of time, while the molecular flux in transwells is time-dependent decreasing gradually from its maximum at the start of the diffusion process to zero at equilibrium when the concentrations at the top and bottom compartments are the same. Therefore, a direct comparison between the permeability results obtained in transwells and microfluidic devices is not appropriate. Instead, it is more appropriate to compare normalized permeability results for each system. The ratio between the permeability with and without cell layers in transwells and microfluidic devices, *P_C_*/*P_N_*, is shown in Figure 10 as a function of molecular size. The trends in both systems are similar as the permeability ratio decreases with increasing molecular size indicating that the paracellular transport is adversely affected as the size of the molecules increases. However, the microfluidics slope is significantly higher than the transwells slope highlighting the additional effect of shear stress. The results suggest that the paracellular-transport dependence on molecular size can be regulated to a certain degree by applying shear stress. Indeed, it is known that TJs form regulated, selectively permeable barriers between two distinct compartments. TJs do not just represent static structural elements but they are dynamically regulated to control paracellular solute and ion transport in diverse physiologic states [53].

While there is a general consensus that shear stress affects paracellular transport, the actual resulting effect is not clear. Shear stress was observed to enhance barrier function in human airway epithelial cells reducing paracellular permeability [54], and the permeability of bovine aortic endothelial monolayers was reported to be enhanced by application of shear [55]. Elsewhere it was suggested that the effect depends on the shear stress level as high shear stress suppresses mitosis and apoptosis while low shear stress supports both processes [51]. Thus, cellular turnover rates and apoptosis rates, and by association the prevalence of leaky junctions and permeability, will be greater in low shear stress regions. In a recent study, permeability was found to increase at the onset of flow and slowly plateaus to a baseline value [27]. Similarly, here the paracellular permeability of smaller molecules is enhanced as the permeability ratio of 4-dextran almost doubled, increasing from about 1/3 in static transwells to about 2/3 in microfluidic devices, due to the applied shear stress.

The permeability results of Lucifer yellow in microfluidic devices are somewhat enigmatic. In transwells, its permeability with either A549 or HUVEC cell monolayer is about half of its permeability without cells, which can be attributed to the effect of paracellular transport similar to the 4-dextran. However, the permeability of Lucifer yellow in microfluidic devices is about the same with and without cell mono or bilayers. While Lucifer yellow has been widely used as a paracellular transport marker, previous work suggested that it could be transported via a transcellular route as well. Cells with organic anion transporters (OATs) may uptake Lucifer yellow since it is anionic in solution. The presence of OATs on A549 epithelial and HUVEC endothelial cells has been reported along with the suggestion that some transcellular transport of Lucifer yellow may occur [56,57]. To determine whether OATs play a significant role in Lucifer yellow molecular transport, a common OAT blocker (probenecid, Santa Cruz Biotechnology, Dallas, TX USA) was used to hinder any OAT activity through competitive inhibition [58]. Confluent A549 monolayers cultured in a transwell top compartment and a microfluidic device top channel were exposed to media solutions mixed with 1 µM Lucifer yellow and 1 µM probenecid. Lucifer yellow concentrations were then measured at the transwell bottom compartment, after two-hour incubation time, and at the device bottom channel outlet, after reaching steady state, similar to previous experiments. 

**Figure 11:**
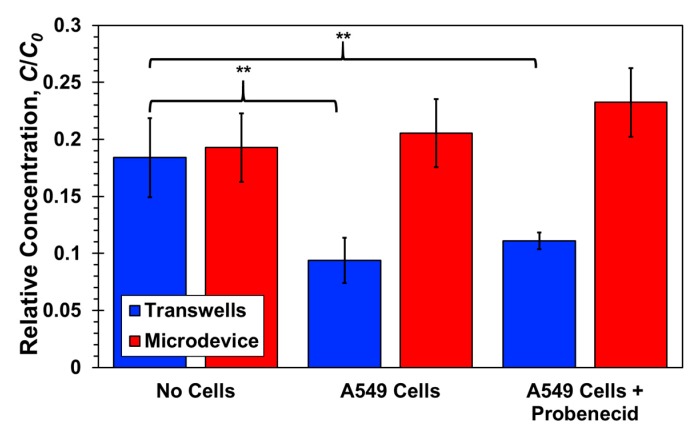
Lucifer yellow relative concentrations with and without a confluent A549 cell monolayer, with and without exposure to probenecid, measured at the bottom compartments of transwells, after a 2-h incubation time, and at the bottom channel outlets of microfluidic devices, under a 20 µL/h flow rate in each channel. Significance determined by Student’s *t*-test; *P < 0.05; **P < 0.01; *n* > 3.

As shown in Figure 11, within experimental error, the measured relative concentrations in both transwells and microfluidic devices are about the same with and without probenecid treatment in presence of a A549 cell monolayer. This may be consistent with more recent reports that OATs are not expressed by HUVEC cells [59,60]. Nevertheless, the contribution of OATs to Lucifer yellow transcellular transport is negligible with or without shear stress. Paracellular seems to be the dominant transport route resulting in reduced permeability through the membrane with confluent A549 monolayer in transwells, *P_C_*/*P_N_* < 1. In microfluidic devices, on the other hand, the Lucifer yellow permeability ratio is about one, *P_C_*/*P_N_* ≅ 1. Thus, the shear stress could only enhance the paracellular transport as observed for 4-dextran, with vanishing effect of the cell monolayer, or augment the enhanced paracellular transport by activating a transcellular transport route other than OATs.

Finally, the permeability ratio for transferrin also increases from *P_C_*/*P_N_* = 0.35 in transwells (Figure 5) to about 0.55 in microfluidic devices (Figure 8). Here again, the role of shear stress in transcellular transport is not very clear. Physiologically laminar shear stress was observed to downregulate the expression of transferrin receptor reducing transcytosis [61], but elevated steady shear stress has also been shown to increase intracellular uptake enhancing endocytosis [62]. Fluid shear stress is known to stimulate both apical and basolateral expression and trafficking of protein transporters [63], which may account for the higher transferrin permeability ratio in microfluidic devices with shear stress.

## 4. Conclusions

Epithelial A549 and endothelial HUVEC cell were successfully co-cultured in a microfluidic membrane bilayer device to model the lung–blood barrier interface. The microfluidic devices enabled characterization of the epithelial/endothelial barrier permeability using measured concentrations resulting because of paracellular and transcellular molecular transport. The bilayer interface permeability for four different molecules was compared with the permeability in microfluidic devices, at steady state, and transwells, after two-hours incubation time, without and with either A549 or HUVEC cell monolayers. The microfluidic transient response reveals that a steady-state molecular distribution is achieved as a balance between convection and diffusion in less than an hour. The molecular flux through the porous membrane in microfluidic devices and transwells, without cells, is inversely proportional to the molecular size because of decreasing diffusivity with increasing size. Paracellular transport of 70-dextran is negligible because of its large size and, in the absence of an active dextran transporter, its slow pinocytosis can also be neglected. As a result, the measured 70-dextran concentrations in microfluidic devices and transwells were within the background noise. Transcellular transport of 4-dextran is similar to that of 70-dextran; however, because of its smaller size, the paracellular transport via tight junctions is not negligible. The permeability of 4-dextran with cells in microfluidic devices and transwells reduces to about two-thirds and one-thirds, respectively, in comparison to the permeability with no cells. The higher ratio in microfluidic devices can be attributed to the flow-induced shear stress known to enhance the leaky tight junctions. Paracellular transport of transferrin is similar to that of 70-dextran; however, because of the expression of transferrin membrane receptors by A549 and HUVEC cells, its transcytosis is significant. Similar to 4-dextran, transferrin permeability ratio in microfluidic devices is higher than in transwells indicating that the transferrin transport pathway may be more active when exposed to shear stress. Lucifer yellow permeability through confluent A549 cell monolayers in transwells and microfluidic devices with and without probenecid treatment, known to block organic anion transporters, is about the same. This suggests that transcellular transport of Lucifer yellow is negligible and, as a small molecule, paracellular is its dominant transport mechanism. Lucifer yellow permeability in transwells with cells is reduced to about half of the permeability without cells. However, in microfluidic devices with shear stress, the permeability with and without cells is about the same.

In summary, the ability to precisely monitor transport properties of bio-species in microfluidic devices with multiple layers of cells, in a more physiological microenvironment, has been demonstrated. Using this technology, we can evaluate the shear stress effect on permeability that cannot be observed in standard static transwell inserts.
